# Phytoplasma Taxonomy: Nomenclature, Classification, and Identification

**DOI:** 10.3390/biology11081119

**Published:** 2022-07-26

**Authors:** Wei Wei, Yan Zhao

**Affiliations:** Molecular Plant Pathology Laboratory, Beltsville Agricultural Research Center, Agricultural Research Service, United States Department of Agriculture, Beltsville, MD 20705, USA; yan.zhao@usda.gov

**Keywords:** phytoplasma, bacterial taxonomy, whole genome-based average nucleotide identity (ANI), *i*PhyClassifier

## Abstract

**Simple Summary:**

Phytoplasmas are vector-borne and graft-transmissible bacteria that cause various plant diseases, leading to severe economic losses. Since phytoplasmas cannot be cultured in cell-free media, their identification and taxonomy rely on molecular techniques and gene sequences. In this article, we summarize the recent advances in phytoplasma taxonomy from three different aspects, including (i) nomenclature (naming *Candidatus* Phytoplasma species); (ii) classification (group and subgroup assignment based on 16S rRNA gene sequences); and (iii) identification (fine differentiation of phytoplasma strains). In addition, some important issues, especially those related to recognizing new ‘*Candidatus* Phytoplasma’ species, are discussed. This information will be helpful for rapid diagnosis of phytoplasma diseases and accurate taxonomic identification of both emerging and known phytoplasma strains.

**Abstract:**

Phytoplasmas are pleomorphic, wall-less intracellular bacteria that can cause devastating diseases in a wide variety of plant species. Rapid diagnosis and precise identification of phytoplasmas responsible for emerging plant diseases are crucial to preventing further spread of the diseases and reducing economic losses. Phytoplasma taxonomy (identification, nomenclature, and classification) has lagged in comparison to culturable bacteria, largely due to lack of axenic phytoplasma culture and consequent inaccessibility of phenotypic characteristics. However, the rapid expansion of molecular techniques and the advent of high throughput genome sequencing have tremendously enhanced the nucleotide sequence-based phytoplasma taxonomy. In this article, the key events and milestones that shaped the current phytoplasma taxonomy are highlighted. In addition, the distinctions and relatedness of two parallel systems of ‘*Candidatus* phytoplasma’ species/nomenclature system and group/subgroup classification system are clarified. Both systems are indispensable as they serve different purposes. Furthermore, some hot button issues in phytoplasma nomenclature are also discussed, especially those pertinent to the implementation of newly revised guidelines for ‘*Candidatus* Phytoplasma’ species description. To conclude, the challenges and future perspectives of phytoplasma taxonomy are briefly outlined.

## 1. Introduction

**Figure 1:**
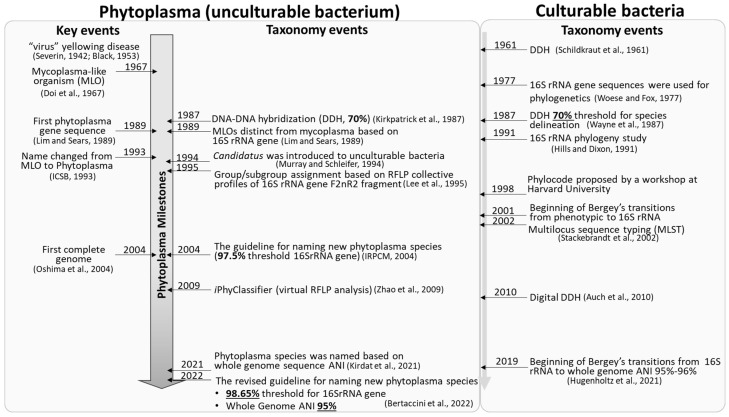
Key events of phytoplasmas and milestones of phytoplasma taxonomy. Details please see references [1,2,3,4,5,6,9,10,11,12,13,14,15,16,17,18,19,20,21,22]. [1] Severin, 1942; [2] Black, 1953; [3] Doi et al., 1967; [4] Lim and Sears, 1989; [5] ICSB, 1993; [6] Oshima et al., 2004; [9] Kirkpatrick et al., 1987; [10] Murray and Schleifer, 1994; [11] Lee et al., 1995; [12] IRPCM, 2004; [13] Zhao et al., 2009; [14] Kirdat et al., 2021; [15] Bertaccini et al., 2022; [16] Schildkraut et al., 1961; [17] Woese and Fox, 1977; [18] Wayne et al., 1987; [19] Hills and Dixon, 1991; [20] Hugenholtz et al., 2021; [21] Stackebrandt et al., 2002; [22] Auch et al., 2010.

Plant diseases characterized by flower abnormality, yellowing, and witches’-broom were long thought to be caused by viruses until 1967 [1,2], when Doi et al. discovered small bacteria with pleomorphism and lack of cell walls in ultrathin electron microscopic sections of infected phloem tissues [3]. The bacteria were named mycoplasma-like organisms (MLOs) because of their morphological resemblance to the mycoplasmas that infect humans and animals [3]. The first 16S rRNA gene sequence of MLO (Oenothera MLO 86-7, accession number M30790) was reported in 1989, which was distinct from mycoplasmas in phylogeny [4]. In 1993, according to the proposal of International Committee on Systematic Bacteriology (ICSB) Subcommittee on the Taxonomy of Mollicutes, the new trivial name, phytoplasma, was used to replace MLO [5]. Phytoplasma is derived from two Greek words, phyto (plant) and plasma (a thing), emphasizing its plant origin. Phytoplasmas have undergone reductive evolution, losing many of the genes involved in metabolic pathways that are essential for free-living organisms [6,7,8]. This not only makes phytoplasmas highly depend on host nutrition, but also is the main reason why axenic culture of phytoplasma has not been achieved yet despite various efforts (Figure 1) [1,2,3,4,5,6,9,10,11,12,13,14,15,16,17,18,19,20,21,22].

Taxonomy (from Greek taxis (arrangement) and nomos (law)) is a broad biological science concerned with nomenclature, classification and identification, which are three related but distinct aspects [23]. Nomenclature is the naming of organisms. Scientific names established according to the binomial nomenclature help scientists around the world better communicate and study the same organism(s). Classification is the orderly arrangement of organisms into groups or taxa based on their similarity. Identification is to recognize a known or unknown organism and to assign it to an existing or a new taxon [23]. Nomenclature is more academic, but classification can be designed according to practical needs [24]. In recent years, significant progress has been made in the bacterial taxonomy; for example, transition from 16S rRNA gene toward whole genome sequence in culturable bacteria [20]. For unculturable phytoplasma, the taxonomy has also been advanced in order to adapt and align better with culturable bacteria. In this article, recent advancements in phytoplasma taxonomy are explored from nomenclature, classification and identification.

## 2. Phytoplasma Nomenclature: Delineation of *Candidatus* Phytoplasma Species

Traditional polyphasic approach, which integrates phenotypic and genotypic data and reflects the ecological nature of the bacteria, is considered as the gold standard for bacterial taxonomy [25]. The phenotypic markers mainly include morphological, physiological, and biochemical characteristics of cultivatable bacteria [26]; however, inability to culture phytoplasma in vitro impeded the accessibility of the above-mentioned phenotypic characteristics to differentiate phytoplasmas. Several decades ago, scientists attempted to distinguish phytoplasmas by using symptoms induced by phytoplasmas, plant host range, insect vector specificity and serological correlations as markers, but were ultimately unsuccessful due to lack of consistency [27,28,29,30,31]. The subsequent development of culture-independent modern genotypic approach based on heredity information has rapidly and considerably enhanced the entire bacterial systematics, providing high levels of resolution and differentiation. In particular, the advent of DNA sequencing technology and exploitation of 16S rRNA gene sequences have tremendously facilitated taxonomy, tree of life, evolution, and diversity studies of unculturable bacteria [32,33,34]. Based on 16S rRNA gene sequences, many bacteria have been reclassified and renamed [35,36].

As with many other unculturable bacteria, the higher rank taxa of phytoplasmas (*Mycoplasmatota* (originally named *Tenericutes*_/*Mollicutes*/*Acholeplasmatales*/*incertae sedis*—Family II]) were named in the absence of type genus and species [37,38,39]. While the *Candidatus* status was used to reserve the putative lower rank taxa (Genus and Species [10]). The term *Candidatus* was first introduced in 1994 to nonculturable bacteria, granting appropriate status of potential taxa based on 16S rRNA gene sequences ([10]; Figure 1). *Candidatus* is not a rank, nor is it governed by Prokaryotic Code [40]. Currently, all phytoplasma strains are accommodated within the provisional *Candidatus* Phytoplasma genus. The main function of the phytoplasma taxonomic nomenclature system is naming ‘*Candidatus* Phytoplasma’ species as species is the most basic taxon of bacteria [12].

### 2.1. Transition from 16S rRNA Gene to Whole Genome-Based Nomenclature of Candidatus Phytoplasma Species?

The first ‘*Candidatus* Phytoplasma’ species (*Ca.* Phytoplasma. aurantifolia) was named based on 16S rRNA gene sequence in 1995 [41]. In 2004, the detailed guidelines (rules *a* through *g*) for naming *Candidatus* Phytoplasma were proposed by the IRPCM Phytoplasma/Spiroplasma Working Team—Phytoplasma taxonomy group [12]. According to the guidelines, *Ca.* Phytoplasma species may be delineated based on the identity of their 16S rRNA gene sequences greater than 1200 bp (rule *a*). A new ‘*Candidatus* Phytoplasma’ species can be recognized if the phytoplasma shares lower than 97.5% sequence identity in 16S rRNA gene with previously established ‘*Candidatus* Phytoplasma’ species (rule *b*). Such sequence identity threshold value was adopted because it corresponded to DNA-DNA hybridization (DDH) reassociation value (70%) suitable for demarcating bacterial species. If a phytoplasma shares higher than 97.5% sequence identity in 16S rRNA gene with existing species but clearly represents “an ecologically separated population”, the phytoplasma also qualifies for a new ‘*Candidatus* species’ (rule *c*).

In genotypic characterization, 16S rRNA gene serves as a backbone for bacterial taxonomy [35,39,40,41,42]. In many bacterial genera, the 16S rRNA gene alone is not sufficient to differentiate species, and, in some cases, multi-locus sequence analysis (MLSA) of alternative housekeeping genes is needed for phylogenetic studies [43,44,45,46]. In addition, with the advancement of genome sequencing technology, whole genome sequence-based genotypic characterization becomes possible. In recent years, the whole-genome average nucleotide identity (ANI) has emerged as a robust method for assessing species boundaries and estimating the genetic relatedness between two genomes. Ample data have shown whole genome ANI is correlated with the traditional microbiological concept of DNA–DNA hybridization relatedness for defining species [47,48]. In bacteria, an ANI value of 95% to 96% has been generally accepted for circumscribing species [47,49]. In 2019, Bergey’s Manual suggested the beginning of the transition from 16S rRNA gene sequence-based to whole genome-based taxonomy in bacteria (Figure 1) [20].

**Figure 2:**
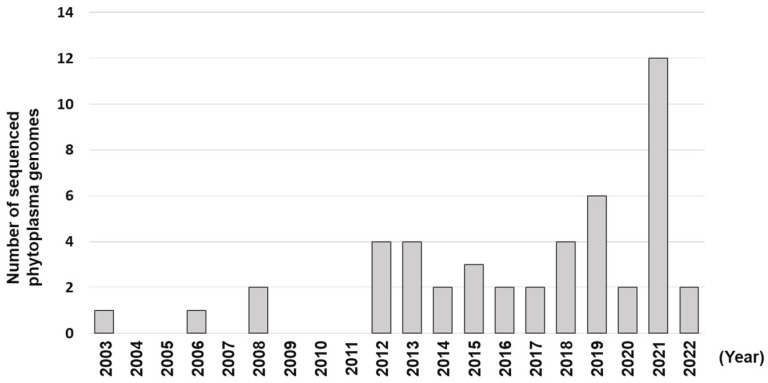
Number of phytoplasma genomes sequenced per year since 2003.

The first complete genome of phytoplasma (onion yellows phytoplasma mild strain (OY-M) was published in 2004 [6] (data deposited into GenBank in 2003). So far, 47 phytoplasma genomes (35 draft and 12 complete) have been sequenced involving 13 groups and 29 subgroups (Figure 2 and Table 1, [6,7,14,50,51,52,53,54,55,56,57,58,59,60,61,62,63,64,65,66,67,68,69,70,71,72,73,74,75,76,77,78,79,80,81]). The size of the complete ge-nomes ranges from 576 to 960 Kb. As shown in Figure 2, 12 phytoplasma genomes were published in year 2021 alone. Even so, genomes of only a small proportion of phytoplasmas have been sequenced compared to nearly one thousand known phytoplasma strains (covering 37 groups and more than 150 subgroups, see Section 3). No doubt, the ever-increasing phytoplasma genome sequence data will serve as an excellent and more comprehensive frame-work for phytoplasma taxonomy.

The 2004 guidelines have served nearly 20 years. To date, approximately 50 ‘*Candidatus* phytoplasma’ species have been formally named [15]. While most of species were delineated based on 16S rRNA gene identity scores (rules *a* and *b*), several species were recognized as they each represent an ecologically distinct population (rule *c*). In contrast, the delineation of ‘*Ca.* Phytoplasma tritici’ and ‘*Ca.* Phytoplasma sacchari’ exploited whole genome information in addition to unique ecological properties [14,82]. Phytoplasma species naming based on whole genome information goes beyond the 2004 guidelines. On the other hand, in recent years, after pairwise comparison of the 16S rRNA gene sequence identity score and the corresponding whole genome ANI score, bacterial taxonomists revised twice the 16S rRNA gene sequence identity threshold value for delineating new bacterial species: changing from 97% to 98.7% and then to the current 98.65% [49,83,84]. To embrace these new developments and to incorporate “whole genome” concept to phytoplasma taxonomy, Bertaccini et al. recently revised guidelines for naming *Ca.* Phytoplasma species (referred to as “2022 guidelines” thereafter) [15].

### 2.2. The Newly Revised 2022 Guidelines and Proposed Amendments

The major revisions in the 2022 guidelines for naming a new *Candidatus* Phytoplasma species include (1) the length of 16S rRNA gene sequence was extended from >1200 bp to >1500 bp (full length or nearly full length of 16S rRNA gene); (2) the threshold of 16S rRNA gene identity was changed from 97.5% to 98.65%; (3) a whole genome ANI criterium was proposed; and (4) if a strain shares >98.65% identity in 16S rRNA gene sequence and >95% genome ANI with previously established species, a MLSA approach can be used to demarcate a new species. Criteria for five housekeeping genes were proposed. However, in our opinion, some provisions in the 2022 guidelines lack clarity and precision. Below, we compare the newly revised 2022 guidelines with the original 2004 guidelines and discuss the issues that require clarification and amendment (Table 2).

In rule (a) of 2004 IRPCM guidelines, the ‘related strain’ was clearly defined. That is, the strain from which this sequence was obtained should be named the ‘reference strain’ and not the ‘type strain’. Strains in which even minimal differences in the 16S rRNA gene sequence from the reference strain are detected do not ‘belong’ to the *Candidatus* species but are ‘related’ to it. However, in the following statement of the 2022 revised guidelines, “*Strains sharing >98.65% sequence identity when compared with the reference strain are considered members of the respective ‘*Ca.* Phytoplasma’ species. Strains showing identity <98.65% to the reference strain, but >98.65% with other strains of the same ‘*Ca.* Phytoplasma’ species should be considered as related to this ‘*Ca.* Phytoplasma’ species.*”.

The term ‘member strain’ was not very well conceived and could lead to erroneous assignment of a single given strain to more than one species. For example, alder yellows phytoplasma strain ALY (AY197646) shares 99.7%, 99.35%, and 98.98% identity with that of ‘*Ca.* Phytoplasma ulmi’ reference strain EY1 (AY197655), ‘*Ca.* Phytoplasma rubi’ reference strain RuS (AY197648), and ‘*Ca.* Phytoplasma ziziphi’ reference strain JWB-G1 (AB052876), respectively, in their 16S rRNA gene sequences. According to the 2022 guidelines, ALY would be a member strain of ‘*Ca.* Phytoplasma ulmi’, ‘*Ca.* Phytoplasma rubi’, and ‘*Ca.* Phytoplasma ziziphi’ simultaneously. Likewise, plum leptonecrosis phytoplasma strain LNp (JQ868450) shares 99.93%, 98.86%, and 98.66% identity with ‘*Ca.* Phytoplasma prunorum’ reference strain ESFY-G1 (AJ542544), ‘*Ca.* Phytoplasma pyri’ reference strain PD1 (AJ542543), and ‘*Ca.* Phytoplasma mali’ reference strain AP15 (AJ542541), respectively, in their 16S rRNA gene sequences. Therefore, according to the 2022 guidelines, LNp would be a member strain of ‘*Ca.* Phytoplasma prunorum’, ‘*Ca.* Phytoplasma pyri’, and ‘*Ca.* Phytoplasma mali’ at the same time.

Conceptually and practically, a strain can be related to more than one species, but cannot be a member of more than one species simultaneously. Therefore, the term “member strain” should be abolished and the term “related strain” as coined in the 2004 original guidelines should be restored.

Rule (c) of the 2004 IRPCM guidelines emphasized the importance of ‘ecological population’; it reflects the ecological nature of bacteria. No matter how we “modernize” our standards or framework, this provision should be retained. In other words, in addition to 16S rRNA sequence identity- and whole ANI-based criteria for demarcating *Candidatus* Phytoplasma species, ecological feature/property-based delineation criteria should be in place as well.

In the 2022 revised guidelines, a proposal was made to allow naming new phytoplasma species based on two out of five housekeeping genes (*groEL*, *tuf*, *rp*, *secA* and *secY*) with individual criteria if a strain shares >98.65% identity in 16S rRNA gene sequence and >95% genome ANI with previously reported species. So far, about 20,000 phytoplasma-related nucleotide sequences have been found in NCBI database, including approximately 8000 16S rRNA gene sequences, 1300 ribosomal protein-encoding gene sequences (*rps*3, *rpl*15, and *rpl*22, etc), 880 *secY* gene sequences, 570 *tuf* gene sequences, and sequences of other genes that are often used for differentiation of closely related phytoplasma strains such as *vmp*, *Cpn60*, *amp*, *map*, and *SecA*, etc. For a particular gene, such as *secY* gene, the sequence length of different phytoplasma strains deposited in GenBank varies considerably, ranging from 150 to 1400 bp. Excessively short sequences bear little value for comparative analysis. In addition, so far, there is no single gene other than the 16S rRNA gene has universal primers that are capable of amplifying all phytoplasma strains. Even widely used generic *rp* and *secY* primers can only amplify phytoplasmas that belong to certain 16Sr groups. Without universal primers and sufficient sequence data for a thorough comparative analysis of these five housekeeping genes, it is still difficult to establish objective criteria for MLSA-based species delineation. However, if the strain clearly represents ecologically separated populations, MLSA could be used to demonstrate significant molecular diversity in addition to fulfilling the unique vectorship and host specificity requirement. In such case, the MLSA assay should not be limited to these five genes proposed in 2022 guidelines. Evidence of significant molecular diversity from other housekeeping genes should be accepted as well.

Based on the above reasoning, we propose the following amendments to the 2022 revised guidelines. For clarity and consistency, the amendments are structured in the same fashion as the original 2004 IRCPM guidelines:

(a) The ‘*Ca.* Phytoplasma’ species description should refer to a single, unique 16S rRNA gene sequence (full length or nearly full length, >1500 bp) or whole genome sequence with at least 60% coverage. The strain from which this sequence was obtained should be named the ‘reference strain’ and not the ‘type strain’. Strains in which even minimal differences in the 16S rRNA gene sequence from the reference strain are detected are referred as ‘related’ to the *Candidatus* species.
(b) In general, a strain can be described as a novel ‘*Ca.* Phytoplasma’ species if its 16S rRNA gene sequence shares <98.65% identity or its whole genome shares an ANI score <95–96% to that of any previously described ‘*Ca.* Phytoplasma’ species.
(c) There are, however, cases of phytoplasmas that share >98.65% identity of their 16S rRNA gene sequences or >95–96% ANI of their genomes, but clearly represent ecologically separated populations and, therefore, may deserve description as separate species. For such cases, description of two different species is recommended only when all three of the following conditions apply:
  (i) the two phytoplasmas are transmitted by different vectors;
  (ii) the two phytoplasmas have a different natural plant host (or, at least, their behavior is significantly different in the same plant host);
  (iii) there is evidence of significant molecular diversity, achieved by either hybridization to cloned DNA probes, serological reaction or multilocus sequence analysis (MLSA).
(d) The rank of subspecies should not be used.
(e) Due to strict international regulations, collection of micropropagation is no longer feasible or realistic; the gene clones of the reference strain should be deposited to the authorized scientific organizations in America, Europe, and Asia, etc. (to be determined by phytoplasma scientists).
(f) Manuscripts that describe a novel ‘*Ca.* Phytoplasma’ species should preferably be submitted to the *Int. J. Syst. Evol. Microbiol* (IJSEM).
(g) The abbreviation for *Candidatus* is *Ca.* (e.g., ‘*Ca.* Phytoplasma japonicum’ stands for ‘*Candidatus* Phytoplasma japonicum’).

In summary, a phytoplasma may be recognized as a novel ‘*Ca.* Phytoplasma’ species if it meets one of the following three criteria: sharing <98.65% 16S rRNA gene sequence identity, or sharing <95–96% genome-wide ANI or representing an ecologically separated population. Fulfillment of the rule (c) shall be demonstrated by vector specificity, unique host or host behavior, and molecular divergence.

## 3. Phytoplasma Classification: 16Sr Group/Subgroup Classification System Based on Collective RFLP Profiles

Classification is the systematic and orderly arrangement of organisms into groups or categories according to established criteria. Different from taxonomic nomenclature system, a classification scheme is often designed to meet practical needs, emphasizing less academic significance. Therefore, different scientists may classify the same organism differently [24]. Phytoplasma classification also has followed this principle. Phenotypic approaches such as symptomology, vectorship, and serology were employed to classify phytoplasmas in early days, but this has proved not suitable or practical [85,86] as in many cases the same phytoplasma strain may induce different symptoms in different hosts, and different phytoplasma strains may share a common vector or cause diseases exhibiting similar symptoms [87]. Until the 1990s, the 16Sr group/subgroup classification scheme was established based on RFLP profiles of PCR amplified F2nR2 fragment of the 16S rRNA gene [11,35,88,89]. This classification system is most widely adopted by phytoplasma researchers so far [90,91,92,93].

The RFLP-based phytoplasma classification scheme exploits a high-resolution subset of the 16S rRNA gene characteristics, namely, the recognition sites of 17 restriction enzymes, to differentiate diverse phytoplasmas [11,87]. The 16Sr groups delineated with this RFLP classification scheme are consistent with the 16S rRNA gene phylogenetic clades. More advantageously, by distinguishing subtle pattern differences, this RFLP analysis-based scheme is able to identify and distinguish different subgroup lineages within any given group [13,88,94,95]. Operationally, traditional RFLP analysis requires actual enzymatic gel electrophoresis and visual comparisons of various banded patterns. It is inconvenient, and few people are willing to do that anymore. The current virtual RFLP analysis approach is operated based on DNA sequences but retains the principles and criteria of the original phytoplasma classification scheme. Using accurate sequence data, the virtual gel patterns generated by computer simulated RFLP analysis can faithfully duplicate the classical and authoritative patterns established by conventional RFLP analysis. The new pattern types derived from virtual RFLP analysis have also been confirmed by actual enzymatic gel electrophoresis [94]. Furthermore, based on the virtual RFLP analysis approach, the interactive online tool *i*PhyClassifier was constructed, enabling and facilitating database-guided phytoplasma classification and identification [13].

Some scientists might think that the RFLP approach is obsolete. The truth is RFLP analysis still plays an important role in the classification and differentiation of many unculturable and fastidious bacteria, and fungi [96,97,98]. Examples include classifications of genus *Basidiobolus* [97] and genus *Vibrio* [98]. In the past five years (2017 to present), around 15,000 papers have been published on the classification and differentiation of bacteria and fungi based on RFLP analysis, including nearly 1600 articles on phytoplasma classification and identification. Computer-simulated virtual RFLP analysis undoubtedly enhanced the applicability of the RFLP analysis-based classification.

Importantly, the 16Sr group/subgroup classification system complements ‘*Candidatus* Phytoplasma’ species affiliation assignment. A striking example is the aster yellows (AY) phytoplasma group, which contains hundreds of known strains around the globe. The current taxonomic system assigns all the AY strains as ‘*Ca.* Phytoplasma asteris’-related strains, which grossly masks the differences among the strains. On the other hand, the existing 16Sr group classification scheme can differentiate the AY strains into more than two dozen subgroups, each of which has its own unique RFLP profile. In addition, some subgroups are only (or predominantly) present in certain geological regions and associated with different ecological niches [93,98].

In addition, in certain cases, the current phytoplasma taxonomic system may even have difficulty to assign certain strains to the existing ‘*Ca.* Phytoplasma’ species. For example, a strain (KJ452548) in the elm yellows phytoplasma group shares 99.1–99.3% identities with ‘*Ca.* Phytoplasma ulmi’- and ‘*Ca.* Phytoplasma ziziphi’-related strains in their 16S rRNA gene sequences. So, what species should this strain be affiliated with, ‘*Ca.* Phytoplasma ulmi’ or ‘*Ca.* Phytoplasma ziziphi’? Well, the RFLP-based group/subgroup classification system can at least provide distinguishing RFLP markers to separate them and classify the strain into a new subgroup other than 16SrV-A and 16SrV-B. This example strongly demonstrates that the group/subgroup classification system effectively avoids the ambiguity caused by the term, ‘*Candidatus* Phytoplasma sp.’-related strain, and helps diagnosticians and regulatory agencies distinguish closely-related phytoplasma strains.

In 2007, based on the virtual RFLP analysis of all 16S rRNA gene sequences available at the time (F2nR2 fragment of about 1250 bp), the number of phytoplasma classification groups was expanded from 19 to 28 (16SrXIX-16SrXXVIII), and some potentially new species were proposed with suggested reference strains (Table 3). In the present review, groups/subgroups corresponding to *Candidatus* Phytoplasma species, especially the newly named species are updated (Table 3 [99,100,101,102,103,104,105,106,107,108,109,110,111,112,113,114,115,116,117,118,119,120,121,122,123,124,125,126,127,128,129,130,131,132,133,134,135,136,137]). Two new groups (16SrXXXVIII and 16SrXXXIX) are established based on the criterium which requires the collective F2nR2 RFLP pattern of any new group representative has a similarity coefficient of <0.85 with that of all previously recognized 16Sr groups [94] (Supplementary Table S1). The reference strains of ‘*Ca.* Phytoplasma noviguineense’ and ‘*Ca.* Phytoplasma dypsidis’ were designated as representative strain of 16SrXXXVIII-A (LC228755) and 16SrXXXIX-A (MT536195), respectively.

Currently, there are a total of 37 groups and 48 named *Candidates* phytoplasma species (Table 3). Each group should contain at least one *Candidatus* species [138]. As shown in Table 3, nearly ten novel groups have been identified since 2007 (16SrXXIX-16SrXXXIX). However, it is noteworthy that no new phytoplasmas have been identified in groups 16SrXXIII-16SrXXVIII during the past 15 years. This suggests that the phytoplasmas belonging to these groups may be rare or the sequences representing these groups contain errors. In addition, we also noted that several pairs of strains share high sequence identity, but very low RFLP similarity coefficients. Such discrepancy might be caused by indels or sequencing errors that occurred within restriction enzyme recognition sites.

## 4. Phytoplasma Identification: Detection, Diagnostics and Characterization

The early identification and diagnosis of phytoplasmas and phytoplasmal diseases are vital for the formulation and implementation of rapid control measures. This not only thwarts the further spread of disease and reduce direct economic losses from plant death/damage, but also prevents delays and restrictions on the import and export of plant materials. Plants infected by phytoplasmas often exhibit remarkable symptoms. These symptoms include virescence (flower petals turning green), phyllody (leafy flowers), cauliflower-like inflorescence (repetitive initiation of inflorescence meristems), and witches’-broom (excessive shoot proliferation) [139,140]. In addition to these characteristic symptoms, phytoplasma infection can also induce some general symptoms seen in diseases caused by various other plant pathogens. Such general symptoms include leaf discoloration (such as purple leaves and leaf yellowing), little leaf, stem fasciation, and stunting [139,140,141]. Furthermore, asymptomatic phytoplasma infections were reported as well [142].

As phytoplasmas cannot be cultured in vitro, the routine culture-dependent metrics and characteristics for bacterial identification (morphological observation, biochemical assay, serotyping and antibiotic inhibition/resistance pattern assessment) cannot be employed. Phytoplasma detection and characterization heavily rely on the molecular diagnostic techniques. With the rapid development of molecular diagnostic techniques, a variety of fast, sensitive, and cost effective phytoplasma detection methods have emerged, ranging from PCR, nested PCR, real time PCR, droplet digital PCR (ddPCR), and loop-mediated isothermal amplification (LAMP) to CRISPR-based detection methods. These methods are devised based on highly conserved gene sequences of phytoplasmas, namely 16S rRNA gene, *rp* gene, *SecY* gene and *tuf* gene, etc. [11,143,144,145,146,147,148,149].

Currently, the most widely adopted procedure for the phytoplasma identification and further classification includes the following steps: (i) PCR or nested PCR amplification of phytoplasma DNA using universal primers of 16S rRNA gene, for example, P1, P7, P1A, P7A, 16S-SR, 16RF2n, and R16R2 [103,144,150,151]; (ii) Sequencing of PCR amplicons (direct sequencing or sequencing after amplicon cloning); and (iii) Sequence analysis using *i*PhyClassifier, classifying the phytoplasma strain under study to existing 16Sr group/subgroup and assigning (relating) the strain to previously named *Candidatus* Phytoplasma species. Results from the last step also offer opportunities for establishing new groups/subgroups and discovering novel *Candidatus* Phytoplasma species.

MLSA-based classification schemes have been established in many bacteria, but not yet implemented in non-culturable phytoplasmas (see Section 2.2 for reasons). However, this does not affect MLSA as a very effective method for phytoplasma diversity studies and fine differentiation of closely related phytoplasmas. For example, MLSA-based approach revealed the genetic diversity of apple proliferation phytoplasmas [152]; in addition, 16S rRNA, *rp*, and *secY* genes based MLSA characterization also indicated azalea little leaf phytoplasmas represented a distinct lineage within 16SrI group [153].

## 5. Challenges and Perspectives

Phytoplasma genome sequence information is essential to further advancing phytoplasma taxonomy. Since axenic phytoplasma culture is unattainable, DNA samples for phytoplasma genome sequencing are usually prepared from infected plants. As host DNA accounts for an overwhelming majority in the genomic DNA preparations, there is a risk of host DNA contamination in the process of genome assembly and mapping. A careful assessment of genome coverage statistics is vital as genome information-based delineation of new *Candidatus* Phytoplasma species solely relies on the accuracy of the genome sequences. Genome coverage statistics can indicate not only the contamination, but also the completeness of the genome. For those incomplete (draft) genomes, a minimum of 60% coverage has been suggested for microbial species delineation [154].

In addition, according to rule (e) in the 2004 Guidelines, the reference strain (maintained in micro-propagation if available) should be sent to and deposited to the scientific community or authorized organizations by the authors of the *Candidatus* species description paper. Implementation of this rule has become increasingly difficult or even no longer feasible due to strict international regulations. A good alternative might be to submit the gene clones of the reference strain to the scientific committee or the authorized organizations in different countries in America, Europe, and Asia, etc. (to be determined by phytoplasma scientists).

Currently, Groups 16SrV, 16SrX, and 16SrXII each contains four or five *Candidatus* Phytoplasma species (Table 3). There are occasions where an unknown strain shares an identical (or nearly identical) 16S rDNA sequence identity score with the reference strains of more than one *Candidatus* species within a given 16Sr group. Such a scenario makes it difficult to determine with which *Candidatus* species the unknown strain should be affiliated. The decision-making process is even tough if any involved *Candidatus* species has quarantine implications. The 2022 Guidelines revised the 16S rRNA gene sequence identity threshold value for demarcating phytoplasma species from 97.5% to 98.65%. The new threshold will likely result in an increase in the number of new *Candidatus* species within certain 16Sr groups, which will make the matters worse. Subgroup classification and multilocus strain typing may help alleviate the problem.

## 6. Conclusions

This article reviews the latest progress in phytoplasma taxonomy from three aspects: nomenclature, classification, and identification. ‘*Candidatus* Phytoplasma’ species/nomenclature system and group/subgroup classification system are two parallel systems and serve different purposes. The nomenclature system focuses more on naming new species based on one of the three criteria: 16S rRNA gene sequence identity (<98.65%), whole genome ANI (<95–96%), or representing ecologically separated populations. Currently, 48 *Candidatus* Phytoplasma species have been named. The group/subgroup classification system is based on collective RFLP profiles of the F2nR2 region of 16S rRNA gene. The genetically diverse phytoplasmas have been classified into 37 groups and more than 150 subgroups.

## Figures and Tables

**Table 1:** A list of complete or draft phytoplasma genomes.

Organism Name	Organism Infraspecific Names Strain	16Sr Group Classification	Host Symptoms	Country	References or GenBank Deposition	Assembly Accession	Assembly Stats Total Sequence Length	Assembly Level	Assembly Submission Date
*‘Catharanthus roseus’* aster yellows phytoplasma	De Villa	I-B	Maize Bushy Stunt-like	South Africa	Coetzee et al. deposited	GCF_004214875.1	603,949	Complete Genome	20 February 2019
*‘Chrysanthemum coronarium’* phytoplasma	OY-V	I-B	onion yellows	Japan	[50]	GCF_000744065.1	739,592	Contig	14 August 2014
*‘Cynodon dactylon’* phytoplasma	LW01	XIV-A	Bermuda grass white leaf	India	[51]	GCF_009268075.1	483,935	Scaffold	22 October 2019
*‘Echinacea purpurea’* witches’-broom phytoplasma	NCHU2014	II-A	purple coneflower witches’ broom	Taiwan	[52]	GCF_001307505.1	545,427	Contig	7 October 2015
*‘Fragaria x ananassa’* phyllody phytoplasma	StrPh-Cl	XIII-F	strawberry phyllody	Chile	[53]	GCF_018274325.1	627,584	Contig	4 May 2021
*‘Parthenium hysterophorus’* phyllody phytoplasma	PR34	II-new subgroup	Santa-Maria phyllody	India	Kirdat deposited	GCF_015100165.1	740,170	Contig	29 October 2020
*‘Parthenium sp.’* Phyllody phytoplasma	PR08	II-D	Santa-Maria phyllody	India	Kirdat deposited	GCF_015239935.1	586,816	Contig	10 May 2021
*‘Santalum album’* aster yellows phytoplasma	SW86	I-B	Sandalwood Spike	India	Tiwarekar deposited	GCF_018283495.1	554,025	Contig	5 May 2021
Aster yellows witches’-broom phytoplasma AYWB	AYWB	I-A	Aster yellows witches’-broom in lettuce	USA	[7]	GCF_000012225.1	723,970	Complete Genome	1 November 2006
*Ca.* Phytoplasma aurantifolia	WBDL	II-C	Lime witches’ broom phytoplasma in periwinkle	Oman	Foissac and Carle deposited	GCF_002009625.1	474,669	Contig	2 March 2017
*Ca.* Phytoplasma luffae	NCHU2019	VIII-A	Loofah witches’ broom	Taiwan	[54]	GCF_018024475.1	769,143	Complete Genome	16 April 2021
*Ca.* Phytoplasma mali	AT	X-A	apple proliferation	NA	[55]	GCF_000026205.1	601,943	Complete Genome	4 July 2008
*Ca.* Phytoplasma	ChTYXIII-Mo	XIII-G	Chinaberry yellowing	Argentina	[56]	GCF_016876135.2	751,949	Contig	14 April 2021
*Ca.* Phytoplasma oryzae	NGS-S10	XI-A	Napier Grass Stunt	Kenya	[57]	GCF_003263355.1	484,488	Contig	25 June 2018
*Ca.* Phytoplasma phoenicium	SA213	XI-D	almond witches’-broom	Lebanon	[58]	GCF_001189415.1	345,965	Contig	30 July 2015
*Ca.* Phytoplasma pini	MDPP	XXI-B	pine phytoplasma	USA	[59]	GCF_007821455.1	474,136	Contig	1 August 2019
*Ca.* Phytoplasma pruni	ChTDIII	III-B	China-tree decline	Argentina	[60]	GCF_013391955.1	790,517	Contig	8 July 2020
*Ca.* Phytoplasma pruni	CX	III-A	Stone fruit tree decline	NA	[61]	GCF_001277135.1	598,511	Contig	1 September 2015
*Ca.* Phytoplasma sacchari	SCGS	XI-B	Sugarcane Grassy Shoot	India	[51]	GCF_009268105.1	505,173	Contig	4 November 2019
*Ca.* Phytoplasma solani	SA-1	XII-A	Bois noir in Periwinkle	NA	[62]	GCF_003698095.1	821,322	Contig	30 October 2018
*Ca.* Phytoplasma solani	284/09	XII-A	Stolbur phytoplasma (in tobacco and parsley)	NA	[63]	GCF_000970375.1	570,238	Chromosome	22 October 2013
*Ca.* Phytoplasma sp. AldY-WA1	AldY-WA1	V-A	Alder yellows	USA	[64]	GCF_020312115.1	457,625	Scaffold	6 October 2021
*Ca.* Phytoplasma tritici	WBD	I-C	00420042pe blue dwarf	China	[65]	GCF_000495255.1	611,462	Contig	1 November 2013
*Ca.* Phytoplasma ziziphi	Jwb-nky	V-B	jujube witches’ broom	China	[66]	GCF_003640545.1	750,803	Complete Genome	12 October 2018
Chrysanthemum yellows phytoplasma	CYP	I-B	Chrysanthemum yellows	Italy	[67]	GCF_000803325.1	659,699	Contig	18 December 2014
Hydrangea phyllody phytoplasma	HP	I-D	Hydrangea phyllody	Japan	[68]	GCF_018327665.1	597,775	Contig	28 April 2021
Italian clover phyllody phytoplasma str. MA1	MA1	III-B	Italian clover phyllody (in periwinkle)	Italy	[69]	GCF_000300695.1	597,245	Contig	1 October 2012
Maize bushy stunt phytoplasma	M3	I-B	Maize bushy stunt	Brazil	[70]	GCF_001712875.1	576,118	Complete Genome	25 August 2016
Milkweed yellows phytoplasma str. MW1	MW1	III-F	Milkweed yellows (in periwinkle)	Italy	[69]	GCF_000309485.1	583,806	Contig	1 October 2012
Mulberry dwarf phytoplasma	MDGZ-01	I-B	Mulberry dwarf	China	[71]	GCF_020714625.1	622,358	Complete Genome	2 November 2021
New Jersey aster yellows phytoplasma	NJAY	I-A	New Jersey aster yellows (in periwinkle)	USA	[72]	GCA_002554195.1	652,092	Contig	16 October 2017
Periwinkle leaf yellowing phytoplasma	DY2014	I-B	Periwinkle leaf yellowing	Taiwan	[73]	GCA_005093185.1	824,596	Contig	2 May 2019
‘Brassica napus’ phytoplasma	TW1	I-new subgroup	Rapeseed stunting and virescence	Canada	[74]	GCA_003181115.1	743,598	Contig	31 May 2018
‘Elaeagnus angustifolia’ witches’-broom phytoplasma	TBZ1	I-new subgroup	Russian olive tree witches’-broom	Iran	Azizpour et al. deposited	GCA_018598675.1	833,199	Contig	30 May 2021
Onion yellows phytoplasma	OY	I-B	Onion yellows (in chrysanthemum)	Japan	[6]	GCA_000009845.1	853,092	Complete Genome	9 December 2003
Paulownia witches’-broom phytoplasma	Zhengzhou	I-D	Paulownia witches’-broom	China	[75]	GCF_019396865.1	891,641	Complete Genome	29 July 2021
Peanut witches’-broom phytoplasma NTU2011	NTU2011	II-A	Peanut witches’-broom (in periwinkle)	Taiwan	[76]	GCF_000364425.1	566,694	Contig	26 March 2013
Poinsettia branch-inducing phytoplasma str. JR1	JR1	III-H	Poinsettia branch-inducing (in periwinkle)	Italy	[69]	GCF_000309465.1	631,440	Contig	1 October 2012
Rice orange leaf phytoplasma	LD1	IX-A	Rice orange leaf	China	[77]	GCF_001866375.1	599,264	Contig	4 November 2016
Sesame phyllody phytoplasma	SS02	II-A or II-D	Sesame phyllody	India	[78]	GCF_018390775.1	536,153	Contig	17 May 2021
*Ca.* Phytoplasma australiense		XII-B	Maintained in periwinkle	Australia	[79]	GCA_000069925.1	879,959	Complete Genome	2 April 2008
Strawberry lethal yellows phytoplasma (CPA)	NZSb11	XII-B variant	Strawberry lethal yellows	Australia and New Zealand	[80]	GCF_000397185.1	959,779	Complete Genome	16 May 2013
Texas Phoenix palm phytoplasma	Flo-TPPD	IV-D	Texas Phoenix Palm decline	USA	Bao et al. deposited	GCF_005774685.1	744,506	Contig	23 May 2019
Vaccinium witches’-broom phytoplasma str. VAC	VAC	III-F	Vaccinium witches’-broom (in periwinkle)	Italy	[69]	GCF_000309405.1	647,754	Contig	1 October 2012
*Ca.* Phytoplasma sp.	Tabriz.2	I-B	*Elaeagnus* sp. (symptoms not described)	Iran	Zirak et al. deposited	GCA_019841745.1	762,261	Contig	24 August 2021
*Ca.* Phytoplasma trifolii-related	CBPPT1	VI-A	Potato purple top (in periwinkle)	USA	Wei et al. deposited	PRJNA839414	514,536	Contig	18 May 2022
Florescence dorée (FD) phytoplasma	CH	V-A	Florescence dorée (in insect vector *Scaphoideus titanus*	Switzerland	[81]	PRJNA838420	654,223	Complete Genome	27 June 2022

**Table 2:** A comparison of the 2022 revised guidelines and the 2004 original guidelines: issues require clarification and amendment.

2004 Guidelines (IRPCM [12])	2022 Revised Guidelines [15]	Suggested Clarification and Amendments to the 2022 Revised Guidelines
**(a)** The ‘*Ca.* Phytoplasma’ species description should refer to a single, unique 16S rRNA gene sequence (**>1200 bp**). The strain from which this sequence was obtained should be named the ‘reference strain’ and not the ‘type strain’. Strains in which even minimal differences in the 16S rRNA gene sequence from the reference strain are detected do not ‘belong’ to the *Candidatus* species, but are ‘related’ to it.	Extended the required length of 16S rRNA gene sequence from >1200 bp to full length or nearly full length. Introduced the term “member strains”. **Comment**: The new term may lead to erroneous assignment of a single given strain to more than one species. See Section 2.2 for details)	**(a)** The ‘*Ca.* Phytoplasma’ species description should refer to a single, unique 16S rRNA gene sequence (**full length or nearly full length, >1500 bp**) or **whole genome sequence with at least 60% coverage** (see Section 4 and Section 5). The strain from which this sequence was obtained should be named the ‘reference strain’ and not the ‘type strain’. Strains in which even minimal differences in the 16S rRNA gene sequence from the reference strain are detected **are referred as ‘related’ to the *Candidatus* species**.
**(b)** In general, a strain can be described as a novel ‘*Ca.* Phytoplasma’ species if its 16S rRNA gene sequence has <**97.5%** similarity to that of any previously described ‘*Ca.* Phytoplasma’ species.	Revised the threshold value for 16S rRNA gene sequence identity-based Ca. Phytoplasma species delineation to 98.65%. Proposed whole genome ANI-based criterum (95%) for Ca. Phytoplasma species delineation.	**(b)** In general, a strain can be described as a novel ‘*Ca.* Phytoplasma’ species if its 16S rRNA gene sequence **shares** <**98.65% identity** or **its whole genome shares an ANI score** <**95–96%** to that of any previously described ‘*Ca.* Phytoplasma’ species.
**(c)** There are, however, cases of phytoplasmas that share >**97.5%** of their 16S rRNA gene sequence, but clearly represent ecologically separated populations and, therefore, may deserve description as separate species. For such cases, description of two different species is recommended only when all three of the following conditions apply: (i) the two phytoplasmas are transmitted by different vectors; (ii) the two phytoplasmas have a different natural plant host (or, at least, their behaviour is significantly different in the same plant host); (iii) there is evidence of significant molecular diversity, achieved by either hybridization to cloned DNA probes, serological reaction or PCR-based assay.	If a strain shares >98.65% similarity in 16S rRNA gene sequence and >95% genome ANI with previously established species, two out of five housekeeping genes (*groEL, tuf, rp, secA* and *secY*) with suggested criteria can be used for delineating new species. **Comment:** All taxonomic frameworks attempt to reflect the ecological nature of organisms. Therefore, Rule (c) of the 2004 guidelines should be retained with necessary modifications. Due to the lack of universal primers and sufficient comparative analysis of housekeeping gene sequence data, it is still difficult to establish objective criteria for species delineation based on MLSA. But if the strain under study clearly represents ecologically separated populations, MLSA could be used to demonstrate significant molecular diversity in addition to fulfilling the unique vectorship and host specificity requirement (see Section 2.2 for details).	**(c)** There are, however, cases of phytoplasmas that share >**98.65%** identity in their 16S rRNA gene sequences or **>95–96% ANI** in their genomes, but clearly represent ecologically separated populations and, therefore, may deserve description as separate species. For such cases, description of two different species is recommended only when all three of the following conditions apply: (i) the two phytoplasmas are transmitted by different vectors; (ii) the two phytoplasmas have a different natural plant host (or, at least, their behaviour is significantly different in the same plant host); (iii) there is evidence of significant molecular diversity, achieved by either hybridization to cloned DNA probes, serological reaction or **MLSA** assay on at least two housekeeping genes.
**(d)** The rank of subspecies should not be used.		**(d)** The rank of subspecies should not be used.
**(e)** The reference strain should be made available to the scientific community from the authors of the *Candidatus* species description paper and it should be deposited (unless in vitro micropropagation proves impossible) in the micropropagated collection of Dr Assunta Bertaccini, DiSTA, Patologia Vegetale, Universita‘ di Bologna, Italy.		**(e) Due to strict international regulations, the rule e may no longer feasible; The gene clones of the reference strain should be deposited to the scientific committee or the authorized organizations in different countries in America, Europe, and Asia, etc (to be determined by phytoplasma scientists).**
**(f)** Manuscripts that describe a novel ‘*Ca.* Phytoplasma’ species should preferably be submitted to the *Int. J. Syst. Evol. Microbiol* (IJSEM).		**(f)** Manuscripts that describe a novel ‘*Ca.* Phytoplasma’ species should preferably be submitted to the *Int. J. Syst. Evol. Microbiol* (IJSEM).
**(g)** The abbreviation for *Candidatus* is Ca. (e.g., ‘*Ca.* Phytoplasma japonicum’ stands for ‘*Candidatus* Phytoplasma japonicum’).		**(g)** The abbreviation for *Candidatus* is Ca. (e.g., ‘*Ca.* Phytoplasma japonicum’ stands for ‘*Candidatus* Phytoplasma japonicum’).

**Table 3:** An updated list of 16Sr groups/subgroups corresponding to named *Candidatus* Phytoplasma species.

Group	Number of ‘*Ca.* Phytoplasma’ Species		Accession Number of Reference Strain	Subgroup	Reference
16SrI: Aster yellows group	3	‘*Ca.* Phytoplasma asteris’	M30790	16SI-B	[99]
		‘*Ca.* Phytoplasma lycopersici’	EF199549	16SrI-Y	[100]
		‘*Ca.* Phytoplasma tritici’	NZ AVAO01000003	16SrI-C	[82]
16SrII: Peanut witches’ broom group	1	‘*Ca.* Phytoplasma aurantifolia’	U15442	16SrII-B	[41]
	* Abolished	‘*Ca.* Phytoplasma australasia’	Y10096	16SrII-D	[101]
16SrIII: X-disease group	1	‘*Ca.* Phytoplasma pruni’	JQ044393	16SrIII-A	[102]
16SrIV: Coconut lethal yellows group	2	‘*Ca.* Phytoplasma palmae’	U18747	16SrIV-A	[12,15]
		‘*Ca.* Phytoplasma cocostanzaniae’	X80117	16SrIV-C	[12,15]
16SrV: Elm yellows group	4	‘*Ca.* Phytoplasma ulmi’	AY197655	16SrV-A	[103]
		‘*Ca.* Phytoplasma ziziphi’	AB052876	16SrV-B	[104]
		‘*Ca.* Phytoplasma rubi’	AY197648	16SrV-E	[105]
		‘*Ca.* Phytoplasma balanitae’	AB689678	16SrV-new subgroup	[106]
16SrVI: Clover proliferation group	2	‘*Ca.* Phytoplasma trifolii’	AY390261	16SrVI-A	[107]
		‘*Ca.* Phytoplasma sudamericanum’	GU292081	16SrVI-I	[108]
16SrVII: Ash yellows group	1	‘*Ca.* Phytoplasma fraxini’	AF092209	16SrVII-A	[109]
16SrVIII: Loofah witches’ broom group	1	‘*Ca.* Phytoplasma luffae’	AF248956	16SrVIII-A	[110]
16SrIX: Pigeon pea witches’ broom group	1	‘*Ca.* Phytoplasma phoenicium’	AF248956	16SrIX-D	[111]
16SrX: Apple proliferation group	4	‘*Ca.* Phytoplasma mali’	AJ542541	16SrX-A	[112]
		‘*Ca.* Phytoplasma pyri’	AJ542543	16SrX-C	[112]
		‘*Ca.* Phytoplasma prunorum’	AJ542544	16SrX-F	[112]
		‘*Ca.* Phytoplasma spartii’	X92869	16SrX-D	[113]
16SrXI: Rice yellow dwarf group	3	‘*Ca.* Phytoplasma oryzae’	AB052873	16SrXI-A	[114]
		‘*Ca.* Phytoplasma cirsii’	KR869146	16SrXI-D	[115]
		‘*Ca.* Phytoplasma sacchari’	VWXM00000000	16SrXI-B	[14]
16SrXII: Stolbur group	5	‘*Ca.* Phytoplasma australiense’	L76865	16SrXII-B	[116]
		‘*Ca.* Phytoplasma japonicum’	AB010425	16SrXII-D	[117]
		‘*Ca.* Phytoplasma fragariae’	DQ086423	16SrXII-E	[118]
		‘*Ca.* Phytoplasma solani’	AF248959	16SrXII-A	[119]
		‘*Ca.* Phytoplasma convolvuli’	JN833705	16SrXII-H	[120]
16SrXIII: Mexican periwinkle virescence group	2	‘*Ca.* Phytoplasma hispanicum’	AF248960	16SrXIII-A	[121]
		‘*Ca.* Phytcoplasma meliae	KU850940	16SrXIII-G	[122]
16SrXIV: Bermudagrass white leaf group	1	‘*Ca.* Phytoplasma cynodontis’	AJ550984	16SrXIV-A	[123]
16SrXV: Hibiscus witches’ broom group	1	‘*Ca.* Phytoplasma brasiliense’	AF147708	16SrXV-A	[124]
16SrXVI: Sugar cane yellow leaf syndrome group	1	‘*Ca.* Phytoplasma graminis’	AY725228	16SrXVI-A	[125]
16SrXVII: Papaya bunchy top group	1	‘*Ca.* Phytoplasma caricae’	AY725234	16SrXVII-A	[125]
16SrXVIII: American potato purple top wilt group	1	‘*Ca.* Phytoplasma americanum’	DQ174122	16SrXVIII-A	[126]
16SrXIX: Japanese chestnut witches’ broom group	1	‘*Ca.* Phytoplasma castaneae’	AB054986	16SrXIX-A	[127]
16SrXX: Buckthorn witches’ broom group	1	‘*Ca.* Phytoplasma rhamni’	X76431	16SrXX-A	[113]
16SrXXI: Pine shoot proliferation group	1	‘*Ca.* Phytoplasma pini’	AJ632155	16SrXXI-A	[128]
16SrXXII: Nigerian coconut lethal decline group	1	‘*Ca.* Phytoplasma palmicola’	KF751387	16SrXXII-A	[129]
16SrXXIII: Buckland Valley grapevine yellows group		1 unnamed species identified	AY083605	16SrXXIII-A	[94]
16SrXXIV: Sorghum bunchy shoot group		1 unnamed new species identified	AF509322	16SrXXIV-A	
16SrXXV: Weeping tea tree witches’ broom group		1 unnamed new species identified	AF521672	16SrXXV-A	
16SrXXVI: Mauritius sugar cane yellows D3T1 group		1 unnamed new species identified	AJ539179	16SrXXVI-A	
16SrXXVII: Mauritius sugar cane yellows D3T2 group		1 unnamed new species identified	AJ539180	16SrXXVII-A	
16SrXXVIII: Havana derbid group		1 unnamed new species identified	AY744945	16SrXXVII-A	
16SrXXIX: Cassia witches’ broom group	1	‘*Ca.* Phytoplasma omanense’	EF666051	16SrXXIX-A	[130]
16SrXXX: Salt cedar witches’ broom group	1	‘*Ca.* Phytoplasma tamaricis’	FJ432664	16SrXXX-A	[131]
16SrXXXI: Soybean stunt phytoplasma group	1	‘*Ca.* Phytoplasma costaricanum’	HQ225630	16SrXXXI-A	[132]
16SrXXXII: Malaysian periwinkle virescence group	1	‘*Ca.* Phytoplasma malaysianum’	EU371934	16SrXXXII-A	[133]
16SrXXXIII: Allocasuarina group	1	‘*Ca.* Phytoplasma allocasuarinae’	AY135523	16SrXXXIII-A	[12]
*16SrXXXIV: grapevine yellows*		No new species identified, abolished	DQ232752		
*16SrXXXV: Pepper witches’-broom*		No new species identified, abolished	EU125184		
16SrXXXVI: foxtail palm yellow decline group	1	‘*Ca.* Phytoplasma wodyetiae’	KC844879	16SrXXXVI-A	[134]
16SrXXXVII: Stylosanthes little leaf group	1	‘*Ca.* Phytoplasma stylosanthis’	MT431550	16SrXXXVII-A	[135]
**16SrXXXVIII: Bogia coconut syndrome group**	**1**	**‘*Ca.* Phytoplasma noviguineense’**	**LC228755**	**16SrXXXVIII-A**	**[136]**
**16SrXXXIX: Palm lethal wilt group**	**1**	**‘*Ca.* Phytoplasma dypsidis’**	**MT536195**	**16SrXXXIX-A**	**[137]**

* Abolished: ‘*Ca.* Phytoplasma australasia’ was originally described by White et al. [101]. It was later removed from the ‘*Ca.* Phytoplasma’ species list by the IRPCM as its 16S rRNA gene sequence shares 99.5% sequence identity with that of ‘Ca. Phytoplasma aurantifolia’ and there is no evidence that it represents an ecologically separated population [12]. ‘*Ca.* Phytoplasma australasia’ was erroneously included in 2022 guidelines [15] and should be removed.

## Data Availability

Not applicable.

## Supplementary Materials

The following supporting information can be downloaded at: https://www.mdpi.com/article/10.3390/biology11081119/s1, Table S1: Identification of two new 16Sr groups/subgroups (16SrXXXVIII-A and 16SrXXXIX-A) based on similarity coefficients derived from virtual RFLP analysis of 16S rRNA genes.
